# Zopiclone and the accuracy of adverse events and mortality reporting

**DOI:** 10.1192/j.eurpsy.2026.12088

**Published:** 2026-07-31

**Authors:** M. Strikić, A. Botica

**Affiliations:** 1 Mental Health Department, Teaching Institute for Public Health; 2 Department of Psychiatry, University Hospital of Split, Split, Croatia

## Abstract

**Introduction:**

Safety reporting is essential due to right clinician’s decision-making. It is a critical part of a healthy environment in clinical trial registration. Zopiclone was first introduced in 1986 in Europe to treat insomnia. Zopiclone itself is not approved in the United States, but its active stereoisomer, eszopiclone, was approved by the FDA in 2024.

**Objectives:**

The main objective of this research was to assess the consistency and transparency of safety reporting of zopiclone in the registry ClinicalTrials.gov and corresponding publications. The categories that were assessed during this research were: Serious Adverse Events, Other Adverse Events, and All-cause mortality. Secondary outcomes of this research were descriptive information provided in the registry fields in the registry and corresponding publications.

**Methods:**

In this cross-sectional study, I followed the Strengthening the Reporting of Observational Studies in Epidemiology (STROBE) reporting guidelines for the reporting. This is a cross-sectional study on RCTs on zopiclone comprising RCTs registered on the ClinicalTrials.gov web registry. We searched ClinicalTrials.gov registry using the term zopiclone, for interventional type of studies, with results posted, registered on or after January 1, 2015, and updated on or before November 1, 2025. For corresponding publications, we searched PubMed, Google Scholar, and Web of Science.

**Results:**

We retrieved 2 trials from ClinicalTrials.gov registry that met our inclusion criteria. We selected eligible RCTs after applying our exclusion criteria. Our exclusion criteria were RCTs that did not study zopiclone, studies without results posted, non- interventional trials, preprints, editorials, letters to editor.

Our findings demonstrated the discrepancies in reporting for 2 RCTs between ClinicalTrials.gov and corresponding publications.

Moreover, the sample size of only two RCTs that met inclusion criteria in the time frame of 10 years in ClinicalTrials.gov demonstrated that the zopiclone was poorly reported in AEs and in corresponding publication. Since ClinicalTrials.gov registry is primarily U.S. we registry it is clear why it is not approved in the United States.

**Conclusions:**

To our knowledge, this is the first study to evaluate the concordance of adverse event reporting from zopiclone in ClinicalTrials.gov and corresponding publications in peer-reviewed journals. Our study assessed discrepancies in adverse events and all-cause mortality reporting from RCTs on zopiclone and presented inconsistent and incomplete reporting in corresponding publications. We found that AE data discrepancies were high between the registry and publications. Discrepancies in the reporting of adverse events from zopiclone trials diminish the transparency of the safety profile of essential sleep disorder treatments.

**Disclosure of Interest:**

None Declared

